# Complete Genome Sequence of an Atypical Porcine Pestivirus Isolated from Jiangxi Province, China

**DOI:** 10.1128/genomeA.00439-18

**Published:** 2018-06-14

**Authors:** SongSong Wu, Zhe Wang, WenBo Zhang, ShunZhou Deng

**Affiliations:** a College of Animal Science and Technology, Jiangxi Agricultural University, Nanchang, Jiangxi Province, China; b Jiangxi Jin Yi Bo Biological Technology Co., Ltd., Nanchang, Jiangxi Province, China

## Abstract

We report here the complete genome sequence of an atypical porcine pestivirus (APPV). The virus strain JX-JM01-2018A01 was isolated from the Jiangxi Province, China, from a sucking piglet. This genome sequence will contribute to the understanding of APPV genetic divergence and promote future disease control and vaccine research and development in China.

## GENOME ANNOUNCEMENT

Atypical porcine pestivirus (APPV) is a novel pestivirus that is the cause of porcine congenital tremor (CT) type A-II, which has been proven by a few reports (1–6). APPV has a distant relationship with classical swine fever virus (CSFV) and bovine viral diarrhea virus (BVDV) (1), and APPV is more likely than CSFV and BVDV to transmit to the next generation of newborn piglets (6). APPV was first reported in North America in 2015 (5, 7), and it can be reproduced by experimental inoculation (5). Furthermore, cases of APPV have been reported in Europe (3, 4, 6, 8, 9) and recently in South America (10), and some reports of APPV in the south of China have also drawn attention to this new emerging porcine infectious disease (1–3, 11).

The isolate JX-JM01-2018A01 was derived from spleen tissue of a sucking piglet exhibiting CT, and the piglet was collected in Yixing City, Jiangxi Province, China, in March 2016. The spleen sample was tested by real-time PCR to determine APPV presence. Total RNA was extracted by using TRIzol reagent (TaKaRa, Dalian, China). The full nucleotide sequences of APPV were amplified by reverse transcription-PCR. The amplified PCR products were purified and inserted into a pUC 19 vector (TaKaRa), and the recombinant plasmid was sequenced with an ABI 3730 genome sequencer (GenScript Company, Nanjing, China). Sequences were assembled and edited by DNAstar Lasergene 15, and the comparative phylogenetic analyses with 10 previous isolates were conducted with MEGA version 7.1 and BioEdit sequence alignment editor with ClustalW software.

The complete genome sequence of isolate JX-JM01-2018A01 is 11,526 bp in length, with a 5' untranslated region (UTR) of 348 nucleotides (nt) and a 3' UTR of 267 nt. A single large open reading frame (ORF) (10,911 nt) was found in the genome between nt positions 349 and 11259, which is capable of coding for a polyprotein of 3,636 amino acids. The comparison between JX-JM01-2018A01 and other APPV sequences available in GenBank revealed the similarity of nucleotide sequences and amino acid sequences. A comparison of the nucleotide identity of JX-JM01-2018A01 with porcine pestivirus 1 (GenBank accession number KY652092), APPV-China/GZ01/2016 (KY475592), NL1 Farm1 (KX929062), AUT-2016_C (KX778724), and Bavaria S5/9 (NC_030653) showed similarities of 93.0%, 92.2%, 90.7%, 90.5%, and 86.4%, respectively. Also, a comparison of amino acid identities showed similarities of 95.2%, 97.2%, 95.6%, 95.4%, and 95.5%, respectively. Genome sequence alignment revealed that our sample is genetically related to APPVs, and phylogenetic analysis grouped this virus into subgenotype 5, indicating that there is a close correlation with the isolates of APPV-China/GZ01/2016 in Guangdong Province and porcine pestivirus 1 in Guangxi Province in China.

To the best of our knowledge, this is the first report of APPV in Jiangxi Province, China. This new genome of APPV from China will provide a better understanding of the genetic diversity and epidemic divergence tendency of APPV. In addition, the new genome will contribute to APPV research for further vaccine development and epidemiological investigations.

### Accession number(s).

The whole-genome sequence of APPV JX-JM01-2018A01 has been deposited in GenBank under the accession number MG792803.
